# Job stress and university faculty members’ life satisfaction: The mediating role of emotional burnout

**DOI:** 10.3389/fpsyg.2023.1111434

**Published:** 2023-02-01

**Authors:** Yin Xu, Yike Wang

**Affiliations:** ^1^School of Foreign Languages, Renmin University of China, Beijing, China; ^2^School of Management, Shanghai University of International Business and Economics, Shanghai, China

**Keywords:** research stress, teaching stress, administrative stress, life satisfaction, emotional burnout

## Abstract

As one of the leading work-related health problems arising from increasingly fierce competition, work-related stress has become a significant predictor of the reduced wellbeing of university faculty members, especially for non-tenured junior faculty members. In light of this and based on a survey, this research seeks to examine how and why work-related stress impacts the life satisfaction level of university junior faculty members. The results indicate that the three subdivisions of university faculty members’ work-related stress, namely, research stress, teaching stress, and administrative stress, are all negatively related to their life satisfaction level. In addition, emotional burnout has been confirmed to function as the psychological mechanism for the aforementioned main effects. The research contributes to the literature mainly by offering a new insight in which the three subdivisions of work-related stress are regarded as independent variables affecting the life satisfaction level of university junior faculty members.

## Introduction

To obtain the benefits of both profitability and sustainability in today’s modern and intensely competitive world, organizations tend to push their employees to the limit (Rana and Soodan, 2019), and academia is no exception (Gentry and Stokes, 2015). Thanks to the prevalence of aggressive pursuit of higher productivity, many universities and academic institutions are blamed for exposing their faculty members to great work-related stress through the *Publish or Perish* policy (Miller et al., 2011; Carvalho and Diogo, 2018), under which university faculty members, especially nontenured junior faculty members, have to function as paper machines to cope with ever-expanding workloads, including publications, patents, research projects, visibility, and teaching (Rana and Soodan, 2019; Griffith and Sovero, 2021). The immoderate work-related stress proves to be the spoiler of university faculty members’ wellbeing, leading to resignations and dismission. According to Inside Higher Ed’s 2022 Survey of College and University Chief Academic Officers, 79% of provosts have confirmed a greater-than-usual faculty turnover rate (Flaherty, 2022). Fidelity Investments and The Chronicle of Higher Education also conducted a survey to find that 69% of university faculty members have shown symptoms of stress and that 55% of faculty members at higher education institutions have seriously considered either changing careers or retiring at an early age (Fidelity Investment and The Chronicle of Higher Education Study, 2021). Moreover, work-related stress is usually associated with increased risks of health problems such as cardiovascular disease (CVD) and affective disorders, making it necessary and urgent to help university faculty members out of work-related stress suffering (Bonde et al., 2009; Nieuwenhuijsen et al., 2010; Steptoe and Kivimäki, 2013). However, worldwide (especially in developing countries), there is no evidence showing any obvious remission of such a prevalent work-related stress attack (Hassard et al., 2018).

Extant literature on work-related stress tends to address topics such as various costs brought by work-related stress [including associated human and organizational costs (e.g., Hassard et al., 2014, 2018), social costs (e.g., Juel et al., 2006; Trontin et al., 2010), substantial economic burdens (e.g., Hoel et al., 2001), causes (e.g., Michie, 2002; Shernoff et al., 2011; Florea and Florea, 2016), effects and impacts (e.g., Elçi et al., 2012; Hung et al., 2012; Sun and Critchfield, 2020), potential solutions (e.g., Dewe and Cooper, 2007; Wickramasinghe, 2010), related policies (e.g., Cousins et al., 2004; Mackay et al., 2004), and management (e.g., Rees, 1995; van der Klink et al., 2001; Arthur, 2004)]. In addition, some researchers tend to discuss work-related stress in terms of a specific occupation. For example, Moustaka and Constantinidis (2010) examined the sources and consequences of work-related stress on the adequacy, productivity, and efficiency of nursing.

However, only a limited number of publications attach importance to how and why work-related stress affects the wellbeing of university junior faculty members. Additionally, the majority of the literature is inclined to treat work-related stress as a general factor while ignoring its specific, concrete subdivisions. Therefore, this research is designed to fill these gaps by determining the exact mechanism through which work-related stress manages to intervene in the wellbeing of university junior faculty members. It is also expected to be among the foregoers to explore the relationship between life satisfaction and three subdivisions of stress (i.e., research stress, teaching stress, and administrative stress).

## Literature review and hypothesis development

### Work-related stress and life satisfaction

Work-related stress is broadly known as a major occupational risk factor attracting much research attention in the field of occupational health psychology (OHP; Kortum et al., 2010; Hassard et al., 2018). Universally defined as a combination of cognitive, subjective, behavioral, and physiological changes resulting from daily occupational affairs, work-related stress constitutes a significant subdivision of the general stress that threatens the wellbeing of most working-class people by exceeding their discretionary resources (Moustaka and Constantinidis, 2010; Wickramasinghe, 2010; Florea and Florea, 2016; Sun and Critchfield, 2020). The literature contributes greatly to research on the causes of work-related stress. According to Dua (1994), work-related stress arises from an individual’s cognitive interpretation of work-related variables (i.e., also known as job stressors), including but not limited to role-related factors such as negligible power, narrow decision latitude, role ambiguity, and role conflict (e.g., Burke, 1988; Nelson and Burke, 2000); bottleneck-related factors such as redundancy threats, undervaluation, and unclear promotion prospects (e.g., Sparks and Cooper, 1999; Yang et al., 2008; Wickramasinghe, 2010); working-environment-related factors such as poor physical working conditions, intense office relationships, and depressive organizational culture (Noblet and Rodwell, 2008); and job-design-related factors such as work overloads, improper working hours, lack of breaks, tough task contents, and task uncertainty (e.g., Michie, 2002; Gold et al., 2006; Florea and Florea, 2016). Moreover, the work-related variables also vary across different occupations, making it necessary to discuss work-related stress in terms of occupation-specific subdivisions. Specifically, the work-related stress of university junior faculty members could be fairly interpreted into research stress, teaching stress, and administrative stress.

Life satisfaction, based on temporarily accessible information that lacks reliability and validity (Schwarz and Strack, 1999), is usually defined as the subjective cognitive evaluation of the quality of someone’s life as a whole (Diener, 1984; Pavot and Diener, 1993; Pavot and Diener, 2008). As an independent and substantial constituent component of subjective well-being (SWB; Diener et al., 1999; Diener and Seligman, 2004; Arthaud-Day et al., 2005), life satisfaction is becoming the new focus of positive psychology research that mainly cares about “the positive end of emotional spectrum” (Pavot and Diener, 2008, p. 137). According to Diener et al. (1985), people’s sense of satisfaction with their life can be assessed by the Satisfaction with Life Scale (SWLS), which has long been the favorite for hundreds of studies to assess life satisfaction as an indispensable component of SWB. In addition, the literature tends to pay special attention to factors modifying the level of life satisfaction, including top-down factors (i.e., personality factors) and bottom-up factors (i.e., situational factors; Heller et al., 2004; Stubbe et al., 2005). It is also noteworthy that work-related stress proves to be a typical example of situational factors (Pavot and Diener, 2008).

### Research stress and life satisfaction

As an academia-specific type of stress that tops the list of university junior faculty members’ work-related stress subdivisions, research stress, also known as research burnout, usually refers to a feeling of mental tension or emotional exhaustion arising from the pressure to accomplish a pile of research tasks in a limited period of time (Singh et al., 2004; Walden and Bryan, 2010; Miller et al., 2011).

Research stress is posited to negatively affect university junior faculty members’ level of life satisfaction. Specifically, the majority of university junior faculty members are not qualified enough to possess a tenure for fair job stability and high academic status. Therefore, the inevitable way for them to acquire professional recognition is to accomplish a series of high-quality research tasks (e.g., publication tasks and grant-seeking tasks) and thus to meet the universities’ performance evaluation criteria (Bird, 2006; Glick et al., 2007). Moreover, the universities’ performance evaluation criteria for junior faculty members are usually demanding in terms of aggressive demands for quantity and quality of publications, scant time allowance, and volatile evaluation standards (i.e., the Publish or Perish policy), making research stress prevalent among university junior faculty members (Miller et al., 2011; Johann, 2022). In other words, university junior faculty members are obliged to take on research stress to obtain a higher academic status and professional recognition. However, the research stress imposed on them turns out to be an obvious source of decreased productivity (Eagan and Garvey, 2015; Berebitsky and Ellis, 2018), which becomes another obstacle preventing the smooth completion of obligatory research tasks. As junior faculty members become trapped in a vicious spiral of “research stress-low productivity-more research stress,” it is even harder for them to improve their academic status, leading to a lower level of life satisfaction (Leung et al., 2000; Beliaeva et al., 2001; Herranz-Bellido et al., 2007).

### Teaching stress and life satisfaction

Teaching stress is also regarded as the main spoiler for the life satisfaction level of university junior faculty members. According to Blix et al. (1994), teaching stress comes from the scarcity of time resources. Specifically, teaching tasks, similar to research tasks, are also compulsory for university junior faculty members who are excluded from the tenure track (Miller et al., 2011; Reevy and Deason, 2014). However, teaching can be incredibly time-consuming, for it not only means giving lectures to students but usually means hours of preparation work as well as a series of evaluative stuff (Sabagh and Saroyan, 2014). In light of this, nontenured junior faculty members have to derive a great part of their time from the precious time allowance intended for research tasks to complete teaching tasks, causing great psychological frustration for failing to make the most of their time (Sirum et al., 2009; Brownell and Tanner, 2012). Additionally, thanks to a lack of teaching experience, junior faculty members tend to be strangers to professional teaching activities that call for ever-innovative teaching strategies, patient guidance, and appropriate interactions with students (Guskey, 2000; Padilla-González and Galaz-Fontes, 2015). As novice teachers, junior faculty members have to waste a large amount of time dealing with teacher-student relationships, the failure of which would make a dent in the level of self-efficacy, thus leading to a decreased level of life satisfaction (Lee et al., 2016).

### Administrative stress and life satisfaction

Administrative stress is believed to be another predictor for the decreased level of junior faculty members’ life satisfaction. According to Soares et al. (2020), administrative tasks refer to a series of activities imposed on university faculty members to improve the visibility of their departments, including but not limited to reach-out meetings, reports writing, and other social activities that have nothing to do with research and teaching (Mancebo, 2007). The reason why such administrative stuff becomes compulsory tasks of university junior faculty members lies in the fact that higher education institutions tend to adopt the Anglo-Saxon model in which universities are seen as business-minded social organizations asking for a dramatic change in the role of university faculty members (Resende, 2005; Mancebo et al., 2006). In light of this, university faculty members, especially nontenured junior faculty members, have to dedicate another part of precious time and effort to dealing with complicated administrative stuff, which impairs their job autonomy and freedom by bringing about a strong sense of being controlled (Leite, 2011). The loss of job autonomy thus leads to a drop in the level of life satisfaction (Schienman, 2002; Nguyen et al., 2003).

*H1:* Research stress negatively affects university junior faculty members’ level of life satisfaction.

*H2:* Teaching stress negatively affects university junior faculty members’ level of life satisfaction.

*H3:* Administrative stress negatively affects university junior faculty members’ level of life satisfaction.

### Work-related stress and emotional burnout

Emotional burnout is defined as a state of psychological depletion caused by prolonged exposure to stressful working environments (Khamisa et al., 2015). As a prevalent multidimensional psychological syndrome (Li et al., 2019), emotional burnout usually consists of three fundamental components, namely, emotional exhaustion, depersonalization, and reduced personal accomplishment (Ofei-Dodoo et al., 2019). According to the literature, one of the instruments that is universally applied in the measurement of emotional burnout is the Maslach Burnout Inventory (MBI), which stands out as a series of its homogeneities because of its simplicity and robustness (Maslach and Jackson, 1981). Specifically, the MBI assesses emotional exhaustion, depersonalization, and reduced personal accomplishment of people in occupational environments by creating a self-report questionnaire with informative psychometric properties (Maslach et al., 2001). In addition, the antecedents of emotional burnout have long been the focus of many researchers. Specifically, factors such as mental overload (Alaimo et al., 2020), high psychological demands (Aguilar et al., 2015), and preventative psychological ownership (Adil and Kamal, 2018) all prove to be significant positive predictors of emotional burnout. It is also noteworthy that incidental factors such as the COVID-19 virus are burnout triggers in all professions, including people-oriented professions (e.g., healthcare, education, and social work) and less people-oriented professions (e.g., athletes; Lin and Huang, 2014). However, university faculty members seem to remain the worst victims of emotional burnout, triggered either by incidental factors or by regular factors (Acosta-Fernández et al., 2019).

### Research stress and emotional burnout

Research stress proves to be a positive predictor of emotional burnout. For one thing, research stress is alternatively known as research burnout, a subdivision of emotional burnout that refers to a negative psychological response toward excessive research workloads imposed on university junior faculty members (Miller et al., 2011). In addition, thanks to their adaptation toward the aggressive evaluation standards imposed on the research performance of university junior faculty members (Bird, 2006), the threshold for self-efficacy has improved to an incredibly high level of psychological demand, thus boosting the level of emotional burnout (León-Rubio et al., 2011; Aguilar et al., 2015).

### Teaching stress and emotional burnout

Teaching stress can also become an important source of university faculty members’ emotional burnout. According to Ofei-Dodoo et al. (2019), teaching tasks not only take up a great part of university junior faculty members’ research time allowance but also derive a certain amount of effort from them to deal with interaction-based activities that are entirely different from analysis-based research activities (David and Quintao, 2012). In other words, junior university faculty have to take on the interference brought by the frequent mixture of interaction-based teaching modes and analysis-based research modes, easily leading to mental overload and thus emotional burnout (Alaimo et al., 2020).

### Administrative stress and emotional burnout

Administrative stress also positively affects the level of emotional burnout. Different from teaching and research tasks, administrative tasks are by nature the byproduct of bureaucracy that has little to do with the practical improvement of university junior faculty members’ academic status or professional recognition (Torelli and Gmelch, 1992; Soares et al., 2020). In light of this, nontenured junior faculty members who have long been exposed to intense competition and the aggressive “publish or perish” policy tend to reckon administrative tasks as redundant and a waste of time, causing a strong sense of low reward and thus expanding emotional burnout (Adil and Kamal, 2018).

*H4:* Research stress increases the level of emotional burnout.

*H5:* Teaching stress increases the level of emotional burnout.

*H6:* Administrative stress increases the level of emotional burnout.

### Emotional burnout and life satisfaction

**Figure 1:**
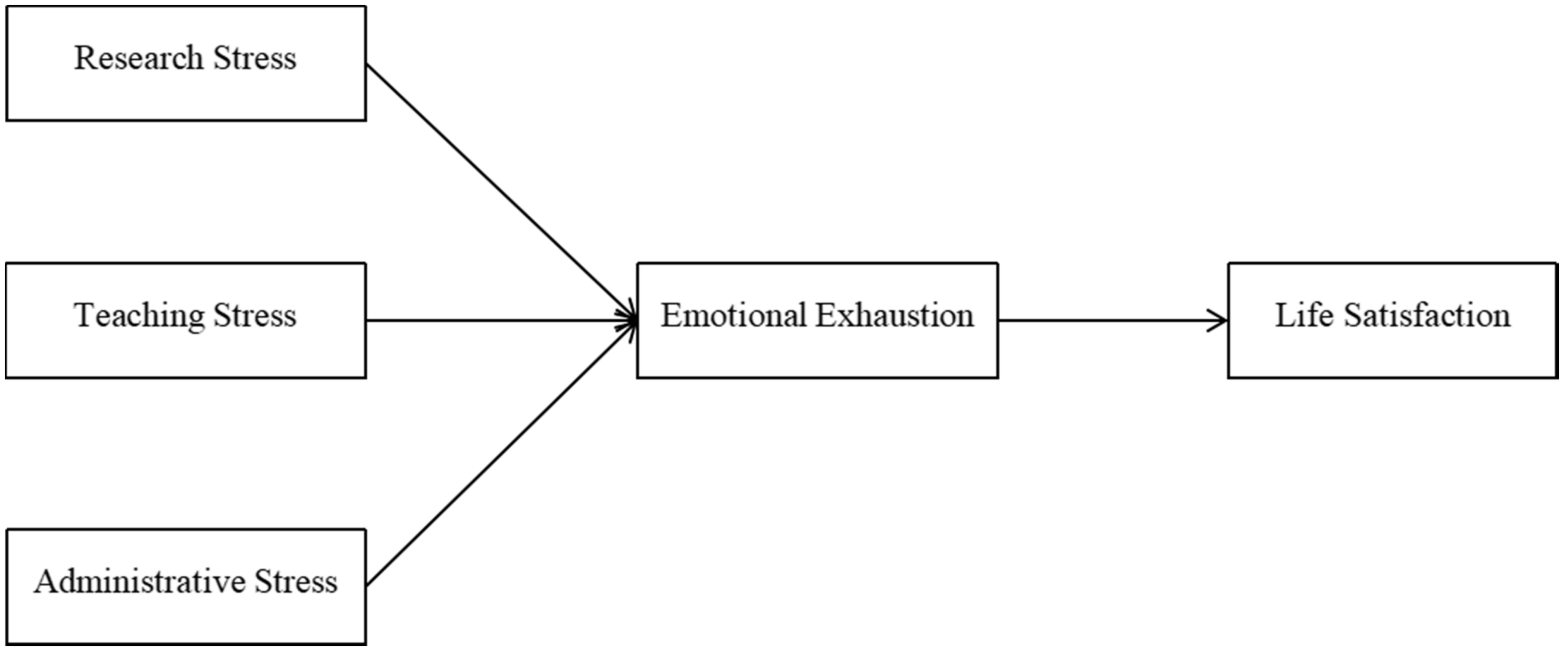
Theoretical model in this study.

There is a warranted negative relationship between university junior faculty members’ emotional burnout and level of life satisfaction because of the health problems brought by it (Khamisa et al., 2015). Every time university faculty members suffer from emotional burnout, it depletes every drop of energy out of their body and thus despoiling the chances of coping with it smoothly (Hobfoll, 1998). In light of this, their body is caught in a combined mess of negative syndromes such as exhaustion, fatigue, somatization and social withdrawal (Gorgievski and Hobfoll, 2008). Moreover, as the physical breakdown brought by long exposure to stressors exhausts the compensatory energy allocated to cope with outside threats, a series of health problems, such as headaches, insomnia, and depression, come to invade the immunity of faculty members’ bodies (Piko, 2006). Therefore, university faculty members’ satisfaction level with life decreases with the deteriorated health state aroused by increased emotional burnout levels (Hobfoll, 2001; Figure 1).

*H7:* Emotional burnout mediates the relationship between research stress and life satisfaction. Specifically, research stress increases the level of emotional burnout, thus decreasing the level of life satisfaction.

*H8:* Emotional burnout mediates the relationship between teaching stress and life satisfaction. Specifically, teaching stress increases the level of emotional burnout, thus decreasing the level of life satisfaction.

*H9:* Emotional burnout mediates the relationship between administrative stress and life satisfaction. Specifically, administrative stress increases the level of emotional burnout, thus decreasing the level of life satisfaction.

## Method

### Sample and procedures

Data were collected using a questionnaire survey. We adopted the snowball sampling method to distribute the questionnaires to university junior faculty members in East China. We invited several junior faculty members to participate in our surveys and asked them to distribute our questionnaires to their colleagues. All participants received a monetary reward after completing the questionnaire. Before sending the questionnaire, we ensured that all the responses would remain anonymous and that the data would only be used for research purposes. This sampling strategy has proven to be a reliable way to collect data (Li et al., 2018). Finally, 202 valid questionnaires were returned, which meets the requirements of our research.

Of this sample, 78 (38.6%) of the respondents were male, and 124 (61.4%) were female. Most of the respondents’ ages were higher than 30 years old (183, 90.6%). With regard to educational level, 138 (68.3%) held a PhD degree. Most (159, 78.7%) of their monthly income was lower than RMB15000.

### Measurements

In this study, all scales were initially studied using English. According to the procedure of Brislin (1970), we translated all the items into Chinese. Certain wordings were adjusted to ensure accuracy and clarity in our research context. All the scales are derived from prior research and have been validated in the Chinese context. All the scales were measured using five-point Likert scales ranging from 1 = strongly disagree to 5 = strongly agree.

#### Research stress

We used the 4-item scale developed by Motowidlo et al. (1986) to measure university junior faculty members’ research stress. Sample items were “My research job is extremely stressful” and “I almost never feel stressed because of my research work” (reverse scored). The Cronbach’s alpha coefficient was 0.94.

#### Teaching stress

We used the 4-item scale developed by Motowidlo et al. (1986) to measure university junior faculty members’ teaching stress. Sample items were “My teaching job is extremely stressful” and “I almost never feel stressed because of my teaching work” (reverse scored). The Cronbach’s alpha coefficient was 0.84.

#### Administrative stress

We used the 4-item scale developed by Motowidlo et al. (1986) to measure university junior faculty members’ administrative stress. Sample items were “My administration job is extremely stressful” and “I almost never feel stressed because of my administration work” (reverse scored). The Cronbach’s alpha coefficient was 0.92.

#### Emotional burnout

We adapted 5-item scale of Schaufeli et al. (1996) to measure university junior faculty members’ emotional burnout. This scale was designed to measure the extent to which participants drained from their work. It includes items such as “I feel burned out from my work” and “Working all day is truly a strain for me.” The Cronbach’s alpha coefficient was 0.92.

#### Life satisfaction

We used life satisfaction scale of Diener et al. (1985) to assess university junior faculty members’ life satisfaction. The scale includes five items that gauge participants’ levels of satisfaction with processes and practices in the workplace. Sample items were “Most days I feel a sense of accomplishment in what I do at work” and “Overall I think I am reasonably satisfied with my life.” The Cronbach’s alpha coefficient was 0.92. Each item was scored on a seven-point Likert scale (1 = “strongly disagree,” 7 = “strongly agree”).

#### Control variables

To better exclude the potential effects of alternative variables, we controlled for four demographic variables, including participants’ gender (coded as 0 = male, 1 = female), age (1 = below 30 years old; 2 = 31–35; 3 = 36–40), education (0 = without a PhD degree; 1 = hold a PhD degree), and monthly income (1 = below RMB5000, 2 = RMB5001–15000; 3 = RMB15001–25000; 4 = above RMB25001).

## Results

To provide preliminary support for the hypotheses, we will first conduct descriptive statistical analyses and correlational analyses among all variables. Following Anderson and Gerbing (1988), we run a series of confirmatory factor analyses (CFAs) to examine the validity of the constructs. We adopted regressions to examine our hypotheses. Moreover, a structural equation model was also conducted to further validate our findings because it can handle complex models (Kline, 2011).

### Preliminary analyses

We conducted CFAs to examine the discriminant validity of our study variables. Table 1 shows the results. Five models were compared in our CFAs: M_0_ represents the null model; M_1_ is our proposed model in which all five variables can be clearly distinguished; M_2_ assumes that emotional burnout cannot be distinguished from life satisfaction; M_3_ examines whether the three kinds of stresses form one factor or they can be clearly distinguished; and finally, M_4_ examines whether the five constructs represent a single indicator. The results of the CFAs can be found in Table 1. According to Table 1, the 5-factor baseline model (M_1_) had the best fit index (*χ*^2^[199] = 371.28, *p* < 0.001; CFI = 0.96, NFI = 0.91, IFI = 0.96, RMSEA = 0.07). Thus, those constructs had good discriminant validity.

**Table 1:** Confirmatory factor analyses of measurement models.

Model specifications	*χ* ^2^	*df*	Δ*χ*^2^	CFI	NFI	IFI	RMSEA
Null model (M_0_)	4065.21	231	–	–	–	–	–
Baseline five-factor model (M_1_)	371.28	199	–	0.96	0.91	0.96	0.07
Emotional burnout and life satisfaction combined (M_2_)	497.84	203	126.56^**^	0.92	0.88	0.92	0.09
Three stresses combined (M_3_)	888.86	206	517.58^**^	0.82	0.78	0.82	0.13
Five constructs represent a single dimension (M_4_)	1555.13	209	1183.85^**^	0.65	0.62	0.65	0.18

*N* = 202. Δ*χ*^2^ is the change of *χ*^2^ compared with the baseline model.

** *p* < 0.01.

The mean value, standard deviation and correlational coefficients of our study variables are shown in Table 2. In terms of the descriptive analysis, university junior faculty members’ research stress (*r* = 0.57, *p* < 0.01), teaching stress (*r* = 0.53, *p* < 0.01) and administration stress (*r* = 0.67, *p* < 0.01) were positively related to emotional burnout. Emotional burnout is negatively correlated with life satisfaction (*r* = −0.82, *p* < 0.01). Interestingly, age (*r* = 0.20, *p* < 0.01) and salary (*r* = 0.49, *p* < 0.01) are positively related to life satisfaction. Together, these results provide preliminary support for our hypothesis.

**Table 2:** Descriptive statistics and correlations among study variables.

	Mean	*SD*	1	2	3	4	5	6	7	8	9
1. Research stress	3.43	1.28	–								
2. Teaching stress	3.28	0.92	0.62^**^	–							
3. Administration stress	2.58	1.14	0.58^**^	0.57^**^	–						
4. Emotional burnout	2.26	1.02	0.57^**^	0.53^**^	0.67^**^	–					
5. Life satisfaction	3.60	1.04	−0.47^**^	−0.43^**^	−0.51^**^	−0.82^**^	–				
6. Gender	0.61	0.49	0.25^**^	0.20^**^	0.08	0.23^**^	−0.25^**^	–			
7. Age	2.36	0.65	−0.10	−0.09	−0.02	−0.17^**^	0.20^**^	−0.04	–		
8. Education	0.68	0.47	0.32^**^	0.37^**^	0.30^**^	0.25^**^	−0.32^**^	0.12	−0.20^**^	–	
9. Monthly income	2.07	0.66	−0.33^**^	−0.32^**^	−0.41^**^	−0.44^**^	0.49^**^	−0.20^**^	0.32^**^	−0.43^**^	–

*N* = 202. Gender: 0 = male, 1 = female; Age: 1 = below 30 years old; 2 = 31–35; 3 = 36–40; Education: 0 = without a PhD degree; 1 = hold a PhD degree; Monthly income: 1 = below RMB5000; 2 = RMB5001-15000; 3 = RMB15001-25000; 4 = above RMB25001.

** *p* < 0.01.

### Hypotheses testing

To test our hypotheses, we adopted multiple regression analyses (Baron and Kenny, 1986; Hox, 2010). The results are shown in Table 3. Hypothesis 1 proposed that university junior faculty members’ research stress negatively affects their life satisfaction. In Model 4, research stress is negatively correlated with life satisfaction (*β* = −0.31, *p* < 0.01). Thus, Hypothesis 1 is supported. Hypothesis 2 proposed that teaching stress negatively affects life satisfaction. In Model 5, university junior faculty members’ teaching stress is not positively correlated with life satisfaction (*β* = −0.28, *p* < 0.01). Thus, Hypothesis 2 is supported. Hypothesis 3 proposed that administrative stress negatively affects life satisfaction. In Model 6, university junior faculty members’ administrative stress is negatively correlated with life satisfaction (*β* = −0.38, *p* < 0.01). Thus, Hypothesis 3 is supported.

**Table 3:** Results of hierarchical multiple regression.

Variables	Emotional exhaustion			Life satisfaction					
	Model 1	Model 2	Model 3	Model 4	Model 5	Model 6	Model 7	Model 8	Model 9
Control variables									
Gender	0.07	0.10	0.16^**^	−0.10	−0.12	−0.16^**^	−0.05	−0.05	−0.03
Age	−0.04	−0.05	−0.12^*^	0.05	0.06	0.10	0.02	0.02	0.01
Education	−0.04	−0.07	−0.04	−0.06	−0.05	−0.06	−0.09	−0.10^*^	−0.09^*^
Monthly income	−0.28^**^	−0.30^**^	−0.14^*^	0.32^**^	0.34^**^	0.24^**^	0.11^**^	0.11^*^	0.13^*^
Independent variables									
Research stress	0.46^**^			−0.31^**^			0.03		
Teaching stress		0.44^**^			−0.28^**^			0.06	
Administration stress			0.61^**^			−0.38^**^			0.11
Mediator									
Emotional burnout							−0.75^**^	−0.76^**^	−0.80^**^
*ΔR^2^*	0.18	0.16	0.30	0.08	0.06	0.12	0.34	0.36	0.31
*ΔF*	57.23^**^	50.26^**^	124.19^**^	24.57^**^	18.21^**^	38.14^**^	214.03^**^	228.54^**^	196.63^**^

*N* = 202. The regression coefficients in the table are all standardized regression coefficients.

* *p* < 0.05;

** *p* < 0.01.

Hypothesis 4 proposed that university junior faculty members’ research stress positively affects their emotional burnout. After controlling for age, gender, salary, and education, in Model 1, research stress is positively correlated with emotional burnout (*β* = 0.46, *p* < 0.01). Thus, Hypothesis 4 is supported. Hypothesis 5 predicted that teaching stress positively affects emotional burnout. Model 2 indicated that teaching stress is positively correlated with emotional burnout (*β* = 0.44, *p* < 0.01). In view of these results, Hypothesis 5 is supported. Hypothesis 6 suggested that administrative stress positively affects emotional burnout. Model 3 showed that administrative stress is positively correlated with emotional burnout (*β* = 0.61, *p* < 0.01). Therefore, Hypothesis 6 is supported.

Furthermore, Hypothesis 7 proposed that emotional burnout mediates the relationship between research stress and life satisfaction. In Model 7, after including junior faculty members’ emotional burnout into the regression, emotional burnout is negatively related to life satisfaction (*β* = −0.75, *p* < 0.01), while the coefficient of research stress becomes nonsignificant (*β* = 0.03, n.s.). Thus, Hypothesis 7 is supported.

Hypothesis 8 proposed that emotional burnout mediates the relationship between teaching stress and life satisfaction. In Model 8, emotional burnout is negatively related to life satisfaction (*β* = −0.76, *p* < 0.01), while the coefficient of teaching stress becomes nonsignificant (*β* = 0.06, n.s.). Hence, Hypothesis 8 is supported.

Finally, Hypothesis 9 proposed that emotional burnout mediates the relationship between administrative stress and life satisfaction. In Model 9, emotional burnout is negatively related to life satisfaction (*β* = −0.80, *p* < 0.01), while the coefficient of administrative stress becomes nonsignificant (*β* = 0.11, n.s.). Based on these results, Hypothesis 9 is supported.

### Path analysis

To further validate our hypotheses, we conducted structural equation modeling. The baseline model is a full mediation model that includes paths from research stress, teaching stress, and administration stress to emotional burnout and a path from emotional burnout to life satisfaction.

**Figure 2:**
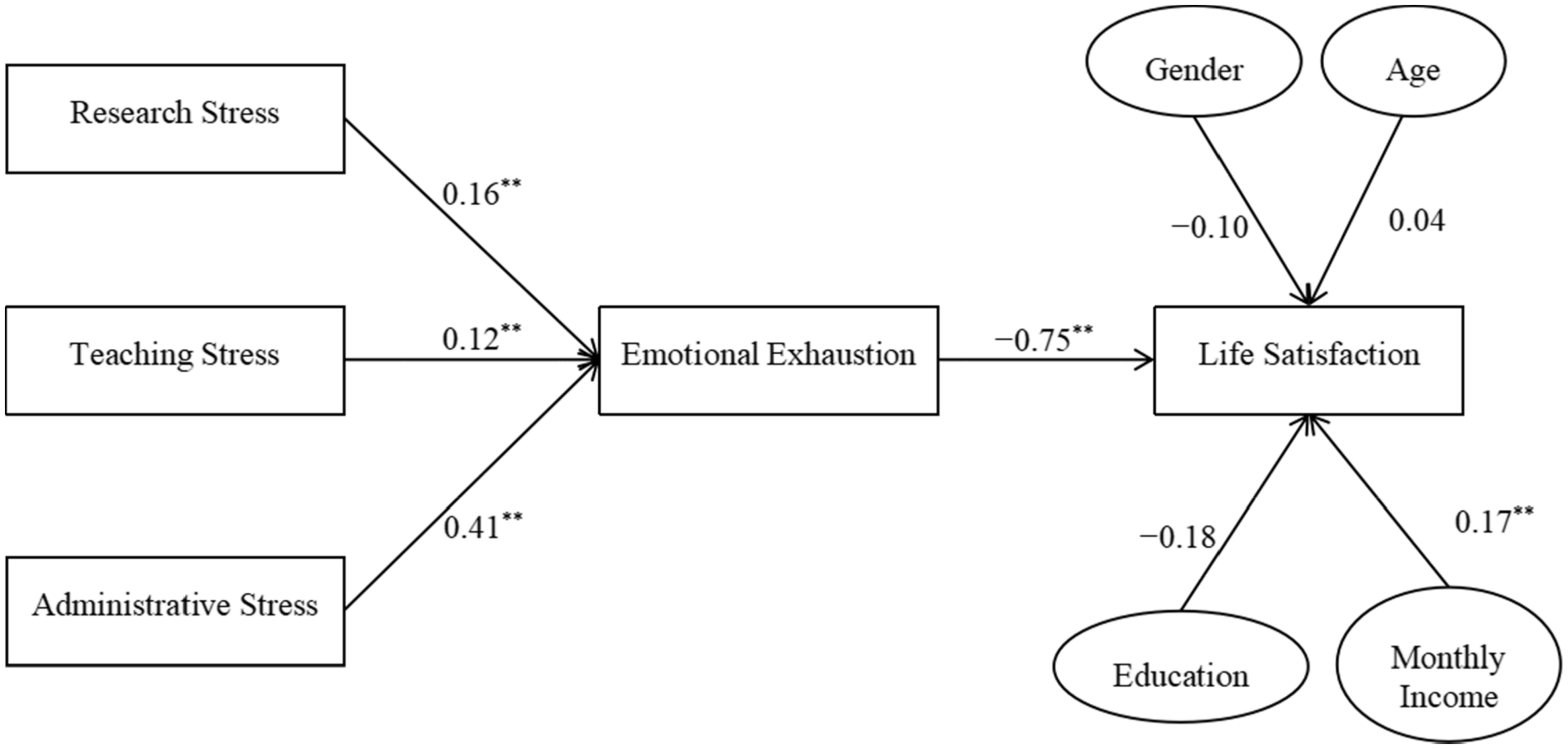
Path analysis coefficients of hypothesized model. **p < 0.01.

Figure 2 shows the path coefficients of this model. All the results are consistent with those in the multiple regression analyses. To further examine Hypotheses 7–9, the mediating effects of emotional burnout were tested for significance using the bootstrapping method (Preacher and Hayes, 2008). As shown in Table 4, the indirect effects of research stress, teaching stress, and administration stress on life satisfaction *via* emotional burnout were significant. Therefore, all the hypotheses were supported.

**Table 4:** Parameter estimates of the mediation model and 95% confidence intervals.

	Estimated effect	95% CI^a^
Direct effects		
Research stress→emotional exhaustion	0.16^**^	[0.06, 0.26]
Teaching stress→emotional exhaustion	0.12^**^	[0.01, 0.24]
Administration stress→emotional exhaustion	0.41^**^	[0.29, 0.53]
Emotional exhaustion→life satisfaction	−0.75^**^	[−0.83, −0.67]
Indirect effects		
Research stress→emotional exhaustion→life satisfaction	−0.12^**^	[−0.20, −0.04]
Teaching stress→emotional exhaustion→life satisfaction	−0.09^**^	[−0.18, −0.01]
Administration stress→emotional exhaustion→life satisfaction	−0.31^**^	[−0.40, −0.21]

*N* = 202.

a CI = confidence interval (1,000 bootstrap samples).

** *p* < 0.01.

## Discussion

Based on a survey, the present paper examined how and why work-related stress impacts the life satisfaction level of university faculty members. The results indicated that the three subdivisions of university faculty members’ work-related stress, namely, research stress, teaching stress, and administrative stress, were all negatively related to life satisfaction. Furthermore, emotional burnout was shown to be the underlying mechanism for the aforementioned effects.

This research contributes to the literature in at least three ways. First, the paper contributes to the expansion of work-related stress research domains by providing insights into how work-related stress affects the life satisfaction level of university junior faculty members, a specific group of university staff that has been overlooked by many researchers. As the literature tends to emphasize the negative relationship between work-related stress and the life satisfaction level of general university faculty members (e.g., Blix et al., 1994; Herranz-Bellido et al., 2007; Miller et al., 2011; Lin and Huang, 2014; Sabagh and Saroyan, 2014; Padilla-González and Galaz-Fontes, 2015; Berebitsky and Ellis, 2018; Soares et al., 2020), the work-related stress faced by university junior faculty members has barely been addressed as a specific research question. Although there exist several publications focusing on nontenured faculty members, who prove to be the largest components of university junior faculty groups, they tend to explore the sources and predictors of nontenured faculty members’ work-related stress rather than its impacts on the level of life satisfaction (e.g., Walden and Bryan, 2010; Reevy and Deason, 2014; Carvalho and Diogo, 2018).

Second, by determining the mediating effect of emotional burnout, this paper explores why work-related stress affects the life satisfaction level of university junior faculty members. Extant literature on work-related stress does show some interest in detecting the underlying mechanism of the negative relationship between work-related stress and life satisfaction (e.g., Extremera et al., 2009; Lee et al., 2016), especially in those publications mainly meant to examine the antecedents of life satisfaction (e.g., Nguyen et al., 2003; Pavot and Diener, 2008), but almost none of them, to the best of my knowledge, has taken into account the uniqueness and particularity of university junior faculty members.

Third, the paper breaks the tradition in which work-related stress is usually regarded as a general variable in most related publications by innovatively treating its three specific subdivisions, namely, research stress, teaching stress, and administrative stress, as the three independent variables affecting the level of university junior faculty members’ life satisfaction. Although the three types of work-related stress have widely been discussed in publications focusing on the specific sources and predictors of work-related stress suffered by university faculty members (e.g., Thorsen, 1996; Thompson and Dey, 1998; Hendel and Horn, 2008; Slade et al., 2016), they have barely been considered as independent variables in empirical studies.

Meanwhile, the present paper also provides practical implications. For example, this study enables university faculty members to correctly identify their sources of tension and comprehend stress. In the near term, university faculty members can improve their mental health levels by using load management (e.g., purposefully and appropriately lessen their workload in terms of research, teaching, and administration). Moreover, it will also help university administrators pay attention to the negative impact of administrative stress on faculty members. Administrators should release some administrative stress to help faculty members focus on high-quality research and teaching outputs.

## Data availability statement

The raw data supporting the conclusions of this article will be made available by the authors, without undue reservation.

## Ethics statement

The studies involving human participants were reviewed and approved by Shanghai University of International Business and Economics. The patients/participants provided their written informed consent to participate in this study.

## Author contributions

YX designed the theoretical framework, worked on the literature review, and drafted the manuscript. YW collected and analyzed the data and drafted the manuscript. All authors contributed to the article and approved the submitted version.

## Conflict of interest

The authors declare that the research was conducted in the absence of any commercial or financial relationships that could be construed as a potential conflict of interest.

## Publisher’s note

All claims expressed in this article are solely those of the authors and do not necessarily represent those of their affiliated organizations, or those of the publisher, the editors and the reviewers. Any product that may be evaluated in this article, or claim that may be made by its manufacturer, is not guaranteed or endorsed by the publisher.

## References

[ref1] Acosta-Fernández M. Parra-Osorio L. Molina C. B. (2019). Occupational stress, burnout, mental health and its relationship with workplace violence in university teachers. Salud. Uninorte. 35, 328–342. doi: 10.14482/sun.35.3.613.62

[ref2] Adil A. Kamal A. (2018). Impact of perceived authentic leadership and psychological capital on burnout: mediating role of psychological ownership. Psychol. Stud. 63, 243–252. doi: 10.1007/s12646-018-0446-x

[ref3] Aguilar S. Howlet P. Diez G. (2015). Burnout syndrome in teachers of a professional institution in Mexico. Publ. Mag. 45, 53–64.

[ref4] Alaimo A. Esposito A. Orlando C. Simoncini A. (2020). Aircraft pilots workload analysis: heart rate variability objective measures and NASA-task load index subjective evaluation. Aerospace 7:137. doi: 10.3390/aerospace7090137

[ref5] Anderson J. C. Gerbing C. (1988). Structural equation modeling in practice: a review and recommended two-step approach. Psychol. Bull. 103, 411–423. doi: 10.1037/0033-2909.103.3.411

[ref6] Arthaud-Day M. L. Rode J. C. Mooney C. H. Near J. P. (2005). The subjective well-being construct: a test of its convergent, discriminant, and factorial validity. Soc. Indic. Res. 74, 445–476. doi: 10.1007/s11205-004-8209-6

[ref7] Arthur A. R. (2004). Work-related stress, the blind men and the elephant. Br. J. Guid. Couns. 32, 157–169. doi: 10.1080/03069880410001692238

[ref8] Baron R. M. Kenny D. A. (1986). The moderator–mediator variable distinction in social psychological research: conceptual, strategic, and statistical considerations. J. Pers. Soc. Psychol. 51, 1173–1182. doi: 10.1037//0022-3514.51.6.1173, PMID: 3806354

[ref9] Beliaeva G. F. Gorshkova I. D. Y. Kostikova I. V. (2001). University women. Russ. Soc. Sci. Rev. 42, 78–99. doi: 10.2753/RSS1061-1428420478

[ref10] Berebitsky D. Ellis M. K. (2018). Influences on personal and professional stress on higher education faculty. J. Prof. 9, 88–110.

[ref11] Bird S. J. (2006). Research ethics, research integrity and the responsible conduct of research. Sci. Eng. Ethics 12, 411–412. doi: 10.1007/s11948-006-0040-9

[ref12] Blix A. G. Cruise R. J. Mitchell B. M. Blix G. G. (1994). Occupational stress among university teachers. Educ. Res. 36, 157–169. doi: 10.1080/0013188940360205

[ref13] Bonde J. P. E. Munch-Hansen T. Wieclaw J. Westergaard-Nielsen N. Agerbo E. (2009). Psychosocial work environment and antidepressant medication: a prospective cohort study. BMC Public Health 9:262. doi: 10.1186/1471-2458-9-262, PMID: 19635130 PMC2728718

[ref14] Brislin R. W. (1970). Back-translation for cross-cultural research. J. Cross-Cult. Psychol. 1, 185–216. doi: 10.1177/135910457000100301

[ref15] Brownell S. E. Tanner K. D. (2012). Barriers to faculty pedagogical change: lack of training, time, incentives, and… tensions with professional identity? CBE Life Sci. Educ. 11, 339–346. doi: 10.1187/cbe.12-09-0163, PMID: 23222828 PMC3516788

[ref16] Burke R. J. (1988). “Sources of managerial and professional stress in large organizations” in Causes, Coping and Consequences of Stress at Work. eds. Cooper C. L. Payne R. (New York, NY: John Wiley & Sons)

[ref17] Carvalho T. Diogo S. (2018). “Non-tenured teachers, higher education,” in Encyclopedia of International Higher Education Systems and Institutions (Dordrecht, Netherlands: Springer). eds. J. C. Shin and P. Teixeira, 1–5.

[ref18] Cousins R. MacKay C. J. Clarke S. D. Kelly C. Kelly P. J. McCaig R. H. (2004). ‘Management standards’ work-related stress in the UK: practical development. Work Stress. 18, 113–136. doi: 10.1080/02678370410001734322

[ref19] David I. C. Quintao S. (2012). Burnout in teachers: its relationship with personality, coping strategies and life satisfaction. Acta Medica Port. 25, 145–155. doi: 10.20344/amp.24, PMID: 23069234

[ref20] Dewe P. Cooper G. L. (2007). “Coping research and measurement in the context of work-related stress” in International Review of Industrial and Organizational Psychology 2007. eds. Hodgkinson G. P. Ford J. K. (West Sussex, UK: John Wiley & Sons Ltd.), 141–191.

[ref21] Diener E. (1984). Subjective well-being. Psychol. Bull. 95, 542–575. doi: 10.1007/978-90-481-2350-6_2 6399758

[ref22] Diener E. Emmons R. A. Larsen R. J. Griffin S. (1985). The satisfaction with life scale. J. Pers. Assess. 49, 71–75. doi: 10.1207/s15327752jpa4901_13 16367493

[ref23] Diener E. Seligman M. E. P. (2004). Beyond money: toward an economy of well-being. Psychol. Sci. Public Interest 5, 1–31. doi: 10.1111/j.0963-7214.2004.00501001.x, PMID: 26158992

[ref24] Diener E. Suh E. M. Lucas R. E. Smith H. L. (1999). Subjective well-being: three decades of progress. Psychol. Bull. 125, 276–302. doi: 10.1037/0033-2909.125.2.276

[ref25] Dua J. K. (1994). Job stressors and their effects on physical health, emotional health, and job satisfaction in a university. J. Educ. Adm. 32, 59–78. doi: 10.1108/09578239410051853

[ref26] Eagan K. J. Garvey J. C. (2015). Stressing out: connecting race, gender, and stress with faculty productivity. J. High. Educ. 86, 923–954. doi: 10.1080/00221546.2015.11777389

[ref27] Elçi M. Şener İ. Aksoy S. Alpkan L. (2012). The impact of ethical leadership and leadership effectiveness on employees’ turnover intention: the mediating role of work related stress. Procedia Soc. Behav. Sci. 58, 289–297. doi: 10.1016/j.sbspro.2012.09.1003

[ref28] Extremera N. Durán A. Rey L. (2009). The moderating effect of trait meta-mood and perceived stress on life satisfaction. Pers. Individ. Differ. 47, 116–121. doi: 10.1016/j.paid.2009.02.007

[ref29] Fidelity Investment and The Chronicle of Higher Education Study (2021). More than half of college and university faculty considering leaving teaching, citing burnout caused by pandemic. Available at: https://newsroom.fidelity.com/press-releases/news-details/2021/Fidelity-Investments--The-Chronicle-of-Higher-Education-Study-More-Than-Half-of-College-and-University-Faculty-Considering-Leaving-Teaching-Citing-Burnout-Caused-by-Pandemic/default.aspx (Accessed October 18, 2022).

[ref30] Flaherty C. (2022). Calling It Quits. Inside Higher Ed. Available at: https://www.insidehighered.com/news/2022/07/05/professors-are-leaving-academe-during-great-resignation (Accessed October 17, 2022).

[ref31] Florea R. Florea R. (2016). Individual and organizational implications of work-related stress. Econ. Transdiscipl. Cogn. 19:28.

[ref32] Gentry R. Stokes D. (2015). Strategies for professors who service the university to earn tenure and promotion. Res. High. Educ. J. 29, 1–13.

[ref33] Glick W. H. Miller C. C. Cardinal L. B. (2007). Making a life in the field of organization science. J. Organ. Behav. 28, 817–835. doi: 10.1002/job.455

[ref01] Gorgievski M. Hobfoll S. D. (2008). “Work can burn us out and fire us up,” in Handbook of stress and burnout in health care, ed. J. R. B. Halbesleben (Nova Science Publishers, Inc.), 7–22.

[ref34] Gold J. E. Park J.-S. Punnett L. (2006). Work routinization and implications for ergonomic exposure assessment. Ergonomics 49, 12–27. doi: 10.1080/00140130500356643, PMID: 16393801

[ref35] Griffith A. L. Sovero V. (2021). Under pressure: how faculty gender and contract uncertainty impact students’ grades. Econ. Educ. Rev. 83:102126. doi: 10.1016/j.econedurev.2021.102126

[ref36] Guskey T. R. (2000). Evaluating Professional Development. Thousand Oaks, CA: Sage.

[ref37] Hassard J. Teoh K. Cox T. Cosmar M. Gründler R. Flemming D. . (2014). Calculating the Costs of Work-Related Stress and Psychosocial Risks: Literature Review. Luxembourg: Publications Office of the European Union.

[ref38] Hassard J. Teoh K. R. H. Visockaite G. Dewe P. Cox T. (2018). The cost of work-related stress to society: a systematic review. J. Occup. Health Psychol. 23, 1–17. doi: 10.1037/ocp0000069, PMID: 28358567

[ref39] Heller D. Watson D. Ilies R. (2004). The role of person versus situation in life satisfaction: a critical examination. Psychol. Bull. 130, 574–600. doi: 10.1037/0033-2909.130.4.574, PMID: 15250814

[ref40] Hendel D. D. Horn A. S. (2008). The relationship between academic life conditions and perceived sources of faculty stress over time. J. Hum. Behav. Soc. 17, 61–88. doi: 10.1080/10911350802165536

[ref41] Herranz-Bellido J. Reig-Ferrer A. Cabrero-García J. Ferrer-Cascales R. González-Gómez J. P. (2007). La satisfacción académica de los profesores universitarios. Actas de las V Jornadas de Investigación en Docencia. Alicante: Universidad de Alicante.

[ref42] Hobfoll S. E. (1998). Stress, Culture and Community: The Psychology and Philosophy of Stress. New York, NY, USA: Plenum Press.

[ref43] Hobfoll S. E. (2001). The influence of culture, community and the nested-self in the stress process: advancing conservation of resources theory. J. Appl. Psychol. 50, 337–421. doi: 10.1111/1464-0597.00062

[ref44] Hoel H. Sparks K. Cooper C. L. (2001). The Cost of Violence/Stress at Work and the Benefits of a Violence/Stress-Free Working Environment. Geneva, Switzerland: International Labour Organization (ILO).

[ref45] Hox J. (2010). Multilevel Analysis: Techniques and Applications. 2nd Edn. New York, NY: Routledge.

[ref46] Hung J. Fisher R. Gapp R. Carter G. (2012). Work-related stress impacts on the commitment of urban transit drivers. J. Manag. Organ. 18, 220–230. doi: 10.5172/jmo.2012.18.2.220

[ref47] Johann D. (2022). Perceptions of scientific authorship revisited: country differences and the impact of perceived publication pressure. Sci. Eng. Ethics 28, 10–25. doi: 10.1007/s11948-021-00356-z, PMID: 35199218 PMC8866300

[ref48] Juel K. Sorensen J. Bronnum-Hansen H. (2006). Risikofaktorer og folkesundhed i Danmark. Copenhagen: Statens Institut for Folkesundhed.

[ref49] Khamisa N. Oldenburg B. Peltzer K. Ilic D. (2015). Work related stress, burnout, job satisfaction and general health of nurses. Int. J. Environ. Res. Public Health 12, 652–666. doi: 10.3390/ijerph120100652, PMID: 25588157 PMC4306884

[ref51] Kline R. B. (2011). Principles and Practice of Structural Equation Modeling. 3rd Edn. New York, NY: Guilford.

[ref52] Kortum E. Leka S. Cox T. (2010). Psychosocial risks and work-related stress in developing countries: health impact, priorities, barriers and solutions. Int. J. Occup. Med. Environ. Health 23, 225–238. doi: 10.2478/v10001-010-0024-5, PMID: 20934955

[ref53] Lee J. Kim E. Wachholtz A. (2016). The effect of perceived stress on life satisfaction: the mediating effect of self-efficacy. Chongsonyonhak Yongu 23, 29–47. doi: 10.21509/KJYS.2016.10.23.10.29, PMID: 27996059 PMC5154683

[ref54] Leite J. L. (2011). As transformações no mundo do trabalho, reforma universitária e seus rebatimentos na saúde dos docentes universitários. Universidade Soc. 48, 84–96.

[ref55] León-Rubio J. M. Cantero F. J. León-Pérez J. M. (2011). Working conditions and differences in the role that selfefficacy plays in the burnout perceived by university staff. An. Psicol. 27, 518–526. doi: 10.1037/t01221-000

[ref56] Leung T. Siu T. Y. Spector P. E. (2000). Faculty stressors and psychological distress among university teachers in Hong-Kong. Int. J. Stress. Manag. 7, 121–138. doi: 10.1023/A:1009584202196

[ref57] Li J. Han X. Wang W. Sun G. Cheng Z. (2018). How social support influences university students’ academic achievement and emotional exhaustion: the mediating role of self-esteem. Learn. Individ. Differ. 61, 120–126. doi: 10.1016/j.lindif.2017.11.016

[ref58] Li Y. Li Y. Castaño G. (2019). The impact of teaching-research conflict on job burnout among university teachers. Int. J. Confl. Manag. 31, 76–90. doi: 10.1108/IJCMA-05-2019-0080

[ref60] Lin S.-H. Huang Y.-C. (2014). Life stress and academic burnout. Act. Learn. High. Educ. 15, 77–90. doi: 10.1177/1469787413514651

[ref64] MacKay C. J. Cousins R. Kelly P. J. Lee S. McCaig R. H. (2004). ‘Management standards’ and work-related stress in the UK: policy background and science. Work Stress. 18, 91–112. doi: 10.1080/02678370410001727474

[ref65] Mancebo D. (2007). Trabalho docente: subjetividade, sobreimplicação e prazer. Psicol. Reflex Crít. 20, 74–80. doi: 10.1590/S0102-79722007000100010

[ref66] Mancebo D. Maués O. Chaves V. L. J. (2006). Crise e reforma do Estado e da Universidade Brasileira: implicações para o trabalho docente. Educare 28, 37–53. doi: 10.1590/S0104-40602006000200004

[ref67] Maslach C. Jackson S. E. (1981). The measurement of experienced burnout. J. Organ. Behav. 2, 99–113. doi: 10.1002/job.4030020205

[ref68] Maslach C. Schaufeli W. B. Leiter M. P. (2001). Job burnout. Annu. Rev. Psychol. 52, 397–422. doi: 10.1146/annurev.psych.52.1.397 11148311

[ref69] Michie S. (2002). Causes and Management of Stress at work. Occup. Environ. Med. 59, 67–72. doi: 10.1136/oem.59.1.67, PMID: 11836475 PMC1740194

[ref70] Miller A. Taylor S. Bedeian A. (2011). Publish or perish: academic life as management faculty live it. Career Dev. Int. 16, 422–445. doi: 10.1108/13620431111167751

[ref71] Motowidlo S. J. Packard J. S. Manning M. R. (1986). Occupational stress: its causes and consequences for job performance. J. Appl. Psychol. 71, 618–629. doi: 10.1037/0021-9010.71.4.618 3804934

[ref72] Moustaka E. Constantinidis T. (2010). Sources and effects of work-related stress in nursing. Health Sci. J. 4, 210–216.

[ref73] Nelson D. L. Burke R. J. (2000). Women executives: health, stress, and success. Acad. Manag. Exec. 14, 107–121. doi: 10.5465/ame.2000.3819310

[ref74] Nguyen A. N. Taylor J. Bradley S., (2003). Job autonomy and job satisfaction: new evidence. Available at: https://eprints.lancs.ac.uk/id/eprint/48658/ (Accessed November 3, 2022).

[ref75] Nieuwenhuijsen K. Bruinvels D. Frings-Dresen M. (2010). Psychosocial work environment and stress-related disorders, a systematic review. Occup. Med. 60, 277–286. doi: 10.1093/occmed/kqq081, PMID: 20511268

[ref76] Noblet A. J. Rodwell J. J. (2008). Integrating job stress and social exchange theories to predict employee strain in reformed public sector contexts. J. Public Adm. Res. Theory 19, 555–578. doi: 10.1093/jopart/mun019

[ref77] Ofei-Dodoo S. Callaway S. P. Engels K. (2019). Prevalence and etiology of burnout in a community-based graduate medical education system: a mixed-methods study. Fam. Med. 51, 766–771. doi: 10.22454/FamMed.2019.431489, PMID: 31596935

[ref78] Padilla-González L. Galaz-Fontes J. F. (2015). “Intention to leave academia and job satisfaction among faculty members: An exploration based on the international CAP survey” in Forming, Recruiting and Managing the Academic Profession. eds. Teichler U. Cummings W. K. (Cham: Springer International Publishing), 225–239.

[ref79] Pavot W. Diener E. (1993). Review of the satisfaction with life scale. Psychol. Assess. 5, 164–172. doi: 10.1007/978-90-481-2354-4_5

[ref80] Pavot W. Diener E. (2008). The satisfaction with life scale and the emerging construct of life satisfaction. J. Posit. Psychol. 3, 137–152. doi: 10.1080/17439760701756946

[ref81] Piko B. F. (2006). Burnout, role conflict, job satisfaction and psychosocial health among Hungarian health care staff: a questionnaire survey. Int. J. Nurs. Stud. 43, 311–318. doi: 10.1016/j.ijnurstu.2005.05.003, PMID: 15964005

[ref82] Preacher K. J. Hayes A. F. (2008). Asymptotic and resampling strategies for assessing and comparing indirect effects in multiple mediator models. Behav. Res. Methods 40, 879–891. doi: 10.3758/brm.40.3.879, PMID: 18697684

[ref83] Rana A. Soodan V. (2019). Effect of occupational and personal stress on job satisfaction, burnout, and health: a cross-sectional analysis of college teachers in Punjab, India. Indian. J. Occup. Environ. Med. 23, 133–140. doi: 10.4103/ijoem.IJOEM_216_19, PMID: 31920263 PMC6941331

[ref84] Rees D. W. (1995). Work-related stress in health service employees. J. Manag. Psychol. 10, 4–11. doi: 10.1108/02683949510081329

[ref85] Reevy G. M. Deason G. (2014). Predictors of depression, stress, and anxiety among non-tenure track faculty. Front. Psychol. 5:701. doi: 10.3389/fpsyg.2014.00701, PMID: 25071667 PMC4085875

[ref86] Resende M. R. S. (2005). Formação e autonomia do professor universitário: um estudo na Universidade Federal de Goiás. PhD dissertation. São Paulo: Pontifícia Universidade Católica.

[ref87] Sabagh Z. Saroyan A. (2014). Professors’ perceived barriers and incentives for teaching improvement. Int. J. Educ. Res. 2, 18–40. doi: 10.12735/ier.v2i3p18

[ref88] Schaufeli W. B. Leiter M. P. Maslach C. Jackson S. E. (1996). “Maslach burnout inventory-general survey” in The Maslach Burnout Inventory. eds. Maslach C. Jackson S. E. Leiter M. P. (Palo Alto, CA: Consulting Psychologists Press)

[ref89] Schienman S. (2002). Socio-economic status, job conditions, and well-being: self-concept explanations for gender-contingent effects. Sociol. Q. 43, 627–646. doi: 10.1111/j.1533-8525.2002.tb00069.x

[ref90] Schwarz N. Strack F. (1999). “Reports of subjective well-being: judgmental processes and their methodological implications” in Well-being: The Foundations of Hedonic Psychology. eds. Kahneman D. Diener E. Schwarz N. (New York: Russell Sage Foundation), 61–84.

[ref91] Shernoff E. S. Mehta T. G. Atkins M. S. Torf R. Spencer J. (2011). A qualitative study of the sources and impact of stress among urban teachers. Sch. Ment. Heal. 3, 59–69. doi: 10.1007/s12310-011-9051-z

[ref92] Singh S. N. Dalal N. Mishra S. (2004). Research burnout: a refined multidimensional scale. Psychol. Rep. 95, 1253–1263. doi: 10.2466/pr0.95.3f.1253-1263, PMID: 15762409

[ref93] Sirum K. L. Madigan D. Kilionsky D. J. (2009). Enabling a culture of change: a life science faculty learning community promotes scientific teaching. J. Coll. Sci. Teach. 38, 197–206. doi: 10.1002/bmb.20364

[ref94] Slade C. P. Ribando R. J. Fortner C. K. (2016). Faculty research following merger: a job stress and social identity theory perspective. Scientometrics 107, 71–89. doi: 10.1007/s11192-016-1881-x

[ref95] Soares M. B. Mafra S. C. T. de Faria E. R. (2020). Factors associated with perceived stress among professors at a federal public university. Rev. Bras. Med. Trab. 17, 90–98. doi: 10.5327/Z1679443520190280, PMID: 32270109 PMC7138496

[ref96] Sparks K. Cooper C. (1999). Occupational differences in the work-strain relationship: towards the use of situation specific models. J. Occup. Organ. Psychol. 72, 219–229. doi: 10.1348/096317999166617

[ref97] Steptoe A. Kivimäki M. (2013). Stress and cardiovascular disease: an update on current knowledge. Annu. Rev. Public Health 34, 337–354. doi: 10.1146/annurev-publhealth-031912-114452 23297662

[ref98] Stubbe J. H. Posthuma D. Boomsma D. I. De Geus E. J. C. (2005). Heritability of life satisfaction in adults: a twin-family study. Psychol. Med. 35, 1581–1588. doi: 10.1017/S0033291705005374, PMID: 16219116

[ref99] Sun W. Critchfield A. J. (2020). Asian Americans’ perceived work-related stress: impacts on job satisfaction and retention. Qual. Res. Rep. 22, 66–79. doi: 10.1080/17459435.2020.1844789

[ref100] Thompson C. J. Dey E. L. (1998). Pushed to margins: sources of stress for African American college and university faculty. J. High. Educ. 69, 324–345. doi: 10.1080/00221546.1998.11775137

[ref101] Thorsen E. J. (1996). Stress in academe: what bothers professors? High. Educ. 31, 471–489. doi: 10.1007/BF00137127

[ref102] Torelli J. A. Gmelch W. H. (1992). Occupational Stress and Burnout in Educational Administration. Available at: https://eric.ed.gov/?id=ED352698 (Accessed November 11, 2022).

[ref103] Trontin C. Lassagne M. Boini S. Rinal S. (2010). Le coût du stress professionnel en France en 2007. Available at: https://www.actineo.fr/sites/default/files/le_cout_du_stress_professionnel_en_france_en_2007.pdf (Accessed October 10, 2022).

[ref104] van der Klink J. J. Blonk R. W. Schene A. H. van Dijk F. J. (2001). The benefits of interventions for work-related stress. Am. J. Public Health 91, 270–276. doi: 10.2105/ajph.91.2.270, PMID: 11211637 PMC1446543

[ref105] Walden P. R. Bryan V. C. (2010). Tenured and non-tenured College of Education Faculty Motivators and Barriers in Grant writing: a public University in the South. J. Res. Admin. 41, 85–98.

[ref106] Wickramasinghe V. (2010). Work-related dimensions and job stress: the moderating effect of coping strategies. Stress. Health 26, 417–429. doi: 10.1002/smi.1314

[ref107] Yang L.-Q. Che H. Spector P. E. (2008). Job stress and well-being: an examination from the view of person–environment fit. J. Occup. Organ. Psychol. 81, 567–587. doi: 10.1348/096317907X243324

